# Supplementary material for: 4D MRI: Robust sorting of free breathing MRI slices for use in interventional settings
Source: PLoS One. 2020 Jun 22;15(6):e0235175. doi: 10.1371/journal.pone.0235175 (PMC7307760; doi:10.1371/journal.pone.0235175)
Supplement: S1 Data — (PDF) [file pone.0235175.s003.pdf]

OTTO-VON-GUERICKE-UNIVERSITÄT MAGDEBURG
Ethik-Kommission
der Otto-von-Guericke-Universität an der Medizinischen Fakultät
und am Universitätsklinikum Magdeburg A.ö.R.
Vorsitzender: Univ.-Prof. Dr. med. C. Huth

Universitätsklinikum • Leipziger Straße 44 • D-39120 Magdeburg

Herrn Prof. Dr. rer.nat. habil. O. Speck
ZENIT
Leipziger Str.44
39120 Magdeburg

Tel.	Fax	eMail	Datum
(0391) 67/14314<br>67/14344	(0391) 67/14354<br>67/290185	norbert.beck@med.ovgu.de<br>ethikkommission@ovgu.de	21.12.2012

Unser Zeichen: **172/12**

Untersuchung von gesunden Probanden bei 3 Tesla zur methodischen Entwicklung von Magnetresonanztomographie-Experimenten

Sehr geehrter Herr Prof. Speck,
sehr geehrte Kolleginnen und Kollegen,

die Ethik-Kommission der Otto-von-Guericke-Universität an der Medizinischen Fakultät und am Universitätsklinikum Magdeburg hat die übergebenen Unterlagen zur o. g. Studie überprüft, in der letzten Kommissionssitzung eingehend erörtert und ist zu der Auffassung gekommen, dass gegen die Durchführung keine ethischen Bedenken bestehen.
Diese **zustimmende Bewertung** ergeht unter dem Vorbehalt gleichbleibender Gegebenheiten.

Die Verantwortlichkeit des jeweiligen Prüfwissenschaftlers / behandelnden Prüfarztes bleibt in vollem Umfang erhalten und wird durch diese Entscheidung nicht berührt. Alle zivil- oder haftungsrechtlichen Folgen, die sich ergeben könnten, verbleiben uneingeschränkt beim Projektleiter und seinen Mitarbeitern.

Beim Monitoring sind die Bestimmungen des Bundes- und Landesdatenschutzgesetzes sowie die sich aus der ärztlichen Schweigepflicht ergebenden Einschränkungen zu beachten, was eine Aushändigung kompletter Patientenakten zum Monitoring ausschließt.
Ein Monitoring personen- und studienbezogener Daten wird dadurch nicht beeinträchtigt.

Um die Übersendung von studienbezogenen Jahresberichten / Abschlussberichten / Publikationen wird unter Nennung unserer Registraturnummer gebeten.

Mit freundlichen Grüßen

(i. A. Dr. med. Norbert Beck, Geschäftsführer)
Prof. Dr. med. C. Huth
Vorsitzender der Ethik-Kommission

Ethik-Kommission
der Otto-von-Guericke-Universität an der Medizinischen Fakultät
und am Universitätsklinikum Magdeburg A.ö.R.
Vorsitzender: Univ.-Prof. Dr. med. C. Huth

Anlage zum Votum der Studie 172/12 vom 21.12.2012

Zum Zeitpunkt der Bewertung der vorstehenden Studie waren folgende Damen und Herren Mitglied der Ethik-Kommission der Otto-von-Guericke-Universität an der Medizinischen Fakultät und am Universitätsklinikum Magdeburg:

Herr<br>Prof. Dr. med. Norbert Bannert	Medizinische Fakultät / Universitätsklinikum, Pädiater
Frau<br>Prof. Dr. phil. Eva Brinkschulte	Medizinische Fakultät / Universitätsklinikum, Bereich Geschichte, Ethik und Theorie der Medizin
Herr<br>Prof. Dr.-Ing. Rolf Findeisen	Fakultät für Elektrotechnik und Informationstechnik, Institut für Automatisierungstechnik
Herr<br>Prof. Dr. med. Christof Huth	Medizinische Fakultät / Universitätsklinikum, Universitätsklinik für Herz- und Thoraxchirurgie
Frau<br>Assessorin Ute Klanten	Medizinische Fakultät / Universitätsklinikum, Stabsstelle Recht
Herr<br>OA Dr. med. Werner Kuchheuser	Medizinische Fakultät / Universitätsklinikum, Institut für Rechtsmedizin
Herr<br>Prof. Dr. rer. nat. Jürgen Läuter	Medizinische Fakultät / Universitätsklinikum, Mathematiker, Biometriker
Herr<br>Prof. Dr. phil. Georg Lohmann	Fakultät Geistes-, Sozial- und Erziehungswissenschaften, Institut für Philosophie
Herr<br>Prof. Dr. med. Frank Peter Meyer	Medizinische Fakultät / Universitätsklinikum, Klinischer Pharmakologe
Herr<br>Prof. Dr. med. Jens Schreiber	Medizinische Fakultät / Universitätsklinikum, Universitätsklinik für Kardiologie, Angiologie und Pneumologie, Fachbereich Pneumologie

Mitglieder der Ethik-Kommission, die in eine Studie eingebunden sind, haben für die Votierung der betreffenden Studie kein Stimmrecht.

Die Ethik-Kommission der Otto-von-Guericke-Universität an der Medizinischen Fakultät und am Universitätsklinikum Magdeburg ist unter Beachtung entsprechender internationaler Richtlinien (ICH, GCP) und nationaler Richtlinien (AMG, GCP-V, MPG, MPKPV) tätig, nach Landesrecht (Hochschulmedizingesetz des Landes Sachsen-Anhalt § 1 Abs. 4, Verordnung über Ethik-Kommissionen zur Bewertung klinischer Prüfungen von Arzneimitteln - Ethik-Kom-VO LSA - i. d. akt. Fassung) legitimiert. Weiterhin besteht eine Registrierung der Ethik-Kommission beim Bundesamt für Strahlenschutz nach § 28g Röntgenverordnung (EK-043/R) und § 92 Strahlenschutzverordnung (EK-046/S) sowie beim Office for Human Research Protections, reg. no. IRB00006099, Rockville, MD, U.S.A..

Dr. med. Norbert Beck
Geschäftsführer der Ethik-Kommission
