# Supplementary material for: 4D MRI: Robust sorting of free breathing MRI slices for use in interventional settings
Source: PLoS One. 2020 Jun 22;15(6):e0235175. doi: 10.1371/journal.pone.0235175 (PMC7307760; doi:10.1371/journal.pone.0235175)
Supplement: Attachment — Submitted filename: Response to Reviewers.pdf [file pone.0235175.s004.pdf]

Response to Reviewers

4D MRI: Robust sorting of free breathing MRI slices for use in interventional settings

24.10.2019

We thank PLOS ONE's academic Editor Enrico Grisan and the anonymous reviewers for their comments. We have attempted to address all remarks in the revised version of the manuscript. Below we give detailed responses to all reviewers comments and describe the corresponding changes in our manuscript.

Enrico Grisan Academic Editor:

01. Please be aware of PLOS ONE policy for making the published research as reproducible as possible, in particular for what it concerns the availability of data. In order for your research to be published, please elaborate on the reasons you are not allowed to share data (even anonymized) and/or the criteria upon which researchers can be granted access.

    We submit the anonymized data set underlying the computed scores as supporting information in a csv file with the revised submission. We are furthermore providing the anonymized MRI image data through the open repository of the university library of the Otto-von-Guericke-University Magdeburg under the creative commons license CC-BY-SA (DOI: 10.24352/UB.OVGU-2019-093; https://doi.org/10.24352/UB.OVGU-2019-093)

Journal Requirements:

02. Please ensure that your manuscript meets PLOS ONE's style requirements, including those for file naming.

    We made sure to follow PLOS ONE's style requirements. In particular, we made sure that figure references follow the style guides when referring to more than one figure at once and made changes to the manuscript accordingly. We made changes so that figures are cited in order of appearance. We checked that all images adhere to the file naming rules of PLOS ONE.

03. We note that you have indicated that data from this study are available upon request. PLOS only allows data to be available upon request if there are legal or ethical restrictions on sharing data publicly.

    Please see our answer to point 01.

04. We note that you received funding from a commercial source: Investitionsbank Sachsen-Anhalt

    We believe there is a misunderstanding. The Investitionsbank Sachsen-Anhalt is an institute of the State of Saxony-Anhalt that has only administrative functions to distribute public funding. It is not a commercial funder.

Reviewer 1

05. The two similarity measures used are referenced by their names in OpenCV - TM_CCORR_NORMED and TM_CCOEFF_NORMED.These measures should be described (mathematically) in the text. They are a key part of the method and discussed at length, but a reader who is unfamiliar with OpenCV software would have no idea what they are. Their exact implementations in OpenCV may even change in the future.

    Thank you for pointing that out. We added mathematical formulas and explanations in the manuscript (cf. page 5 lines 147-153 and page 7 lines 210-212) to make the used similarity measures more transparent to readers.

06. There are a number of grammatical / spelling errors in the text which should be corrected. For example:
    a. the sentence beginning on line 5 should read "Our application scenario[s] are ... where the challenge of a moving target exist[s] ... but none satisfy all [the] needs ..." (corrections in [])

       Thank you for pointing that out. We corrected the sentence.

    b. the sentence beginning on line 108 seems to have had some words cut out of it

       Thank you for pointing that out. We corrected the sentence.

    c. the caption for Figure 6 misspells 'contrast'

       Thank you for pointing that out. We corrected the sentence.

    We also have had the revised manuscript proofread by a native speaker.

Regarding the data availability, it is reasonable to withhold the actual MRI image data - the journal suggests though that the numerical scores used to generate the summary statistics in, for example, tables 2 and 3 could be provided.

Thank you for that point. Please see our answer to point 01.

Reviewer 2

07. Please contextualize the imaging time (40 to 80 minutes) for interventional imaging. For non-interventionists, this seems like a very long acquisition time.

    Thank you for pointing that out. We made changes to the manuscript (cf. page 4 lines 105 - 118) to better point out how the total acquisition time is to understand. See lines 107 to 122 in the revised manuscript with marked-up changes.

08. Please comment on why only healthy volunteers were scanned and speculate on what limitations the method may face in the presence of irregular breathing that is more likely in a patient population.

    Thank you for pointing that out. We changed the manuscript (cf. page 10 lines 322 - 330) to address this question. See lines 335 to 343 in the revised manuscript with marked-up changes.

09. Please briefly describe TM CCOEFF NORMED and TM CCORR NORMED

Thank you for pointing that out. Please see our answer to point 05.

10. Please comment on the limitations of manual steps such as ROI selection in the scanner environment. Could such steps be automated?

Thank you for pointing that out. We changed the manuscript (cf. page 10 lines 331 – 338) to address the limitations of manual steps in our method. See lines 344 to 351 in the revised manuscript with marked-up changes.

11. Please provide reconstruction times in the body of the paper.

Thank you for pointing that out. We changed the manuscript (cf. page 9 lines 272 - 274) to provide mean reconstruction times of both methods as well as the respective standard deviations. See lines 284 to 286 in the revised manuscript with marked-up changes.
